# Disparities in Representation of Asian Participants and Investigators in Cardiometabolic Trials∗

**DOI:** 10.1016/j.jacasi.2023.06.006

**Published:** 2023-08-08

**Authors:** Kavita Singh, Dorairaj Prabhakaran

**Affiliations:** a Public Health Foundation of India, Gurugram, Haryana, India, and Heidelberg Institute of Global Health, Heidelberg University, Heidelberg, Germany; b Centre for Chronic Disease Control, Public Health Foundation India, New Delhi, India; c London School of Hygiene & Tropical Medicine, London, United Kingdom

**Keywords:** cardiometabolic, clinical trials, diversity, race

**Figure:**
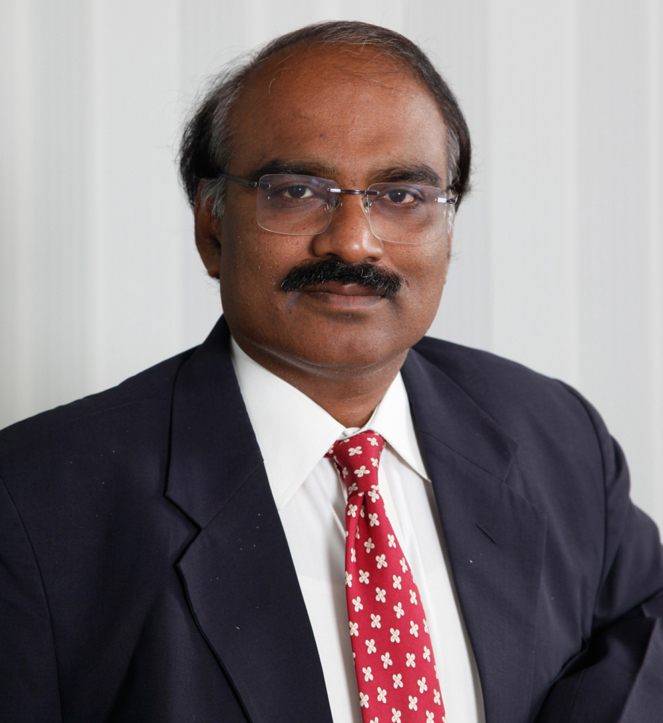

Randomized controlled trials generate the most robust evidence on the safety and effectiveness of health care interventions and inform future research and funding priorities. However, the noninclusion of a diverse range of research participants (age, gender, ethnicity, comorbidities, socioeconomic status), leads to limited generalizability (external validity) of trial findings to the real world. Although efforts by various regulatory bodies and funders toward the inclusion of diverse participants remain at the forefront of clinical research policy, the improvements are marginal, particularly for chronic conditions. Cardiometabolic diseases (CMD) are on the rise globally. People from low-middle-income countries are at an increased risk of developing CMD, but receive suboptimal treatment with fatal outcomes.^1^ Such disparities in care gaps are often referred to as the 80:20 gap, meaning that 80% of the global population at higher risk of developing CMD lives in developing countries, but receives <20% of recommended treatment.^2^^,^^3^ And <10% of global health expenditures are spent on CMDs in low-middle-income countries.^4^

An important factor contributing to the health disparities in low-middle-income countries may be related to the limited knowledge regarding what treatment works best for the geographically and ethnically diverse population at high risk of CMD, which is further aggravated by under-reporting and under-representation of diverse ethnic groups. The paucity of diversity in clinical trial participants generates a race/ethnic data gap that skews medical evidence and innovation toward therapies with understudied efficacy and safety for underserved populations. This disparity contributes to biased evidence and excludes people with unmet medical needs from the benefits of clinical trial participation. Previous studies found widely varying levels of race or ethnicity reporting and representation.^5^, ^6^, ^7^ Few had sufficient size or scope to permit investigation of trends over time. Those who did investigate changes over time found inconsistent trends. Studies also found differing associations between industry and governmental funding. Studies seldom conducted multivariable analyses to control for other factors. The divergent methods and sampling strategies among studies prevent effective comparisons between trial characteristics and the representation of diverse populations. Particularly, little is known about the representation of Asians/Asia Pacific (APAC) region in seminal CMD trials published in high-impact medical journals.

In this issue of *JACC: Asia*, Azzopardi et al^8^ analyzed the proportion and trends of APAC region authorship within high-impact cardiometabolic trials from 2011 to 2020. The authors performed a systematic review of all cardiovascular, diabetes and obesity-related randomized controlled trials (trial phase, ≥2; number of participants, ≥100) published in the *New England Journal of Medicine*, *The Lancet*, and *JAMA* between January 2011 and December 2020. Further, the authors examined the temporal trends using the Jonckheere–Terpstra proportion test and correlations using Pearson’s correlation coefficient and calculated participant-to-prevalence ratios using Global Health Data Exchange registry data. The authors found that, in 656 cardiometabolic trials from *JAMA*, the *New England Journal of Medicine*, and *The Lancet*, only 8.3% of participants were of Asian race, 7.9% of lead authors, and 10.0% of collaborators (co-authors) were from the APAC region. A marginal increase in the proportion of Asian race between 2011 and 2020 (Δ1.40% ± 6.9% per year; *P* = 0.003) and APAC regional (Δ1.46% ± 8.6% per year; *P* = 0.003) enrolment was observed; however, a severe regional under-representation persisted (population prevalence ratio, <0.3). Further, most of the APAC region’s lead authors were from Australia, China, Korea, Japan, and India.

The disheartening statistics (<1 in 12 patients enrolled from APAC) from this study have implications for clinical research, practice, and policy decisions. With more than 60% of the global burden of CMD residing in APAC, this represents a severe under-representation of patients from this region who have unmet treatment needs. Industry-funded trials had a significantly lower representation of lead authors from APAC, but enrolled slightly better diverse participants than government-funded trials. Further, the study revealed a modest increase in the lead authorship and enrollment of Asian/APAC participants in trials, however, only in selected countries (Oceania and East Asia); there were no difference in South Asia or Southeast Asia. Positive trends in both reporting and representation over time may reflect the impact of the various initiatives by regulatory authorities to improve diversity in recruitment. APAC-led and -authored trials had a greater proportion of APAC recruitment, suggesting a concerted effort to create local capacity and scientific leadership in this region would improve Asian enrolment.

However, findings from this study should be interpreted with careful consideration. The review considered only trials published in 3 top journals as a proxy of trial impact. Still, we recognize that increasingly even very high-quality, well-designed, and well-conducted trials of clinical and regional importance are sometimes declined at the editorial desk review stage and transferred to other specialty journals that may have underestimated APAC trial participants and authorship representation. Although studies published in top-tier journals serve as a proxy of high-impact work, these medical journals situated in North America or Europe may be more receptive or inclined to disseminate findings of locally conducted trials in the United States or Europe. Also, sometimes the local funding bodies in Asia/APAC region mandate the publication of the trial results in open-access national medical journals to enhance the impact of local research and for wider dissemination of trial results among local stakeholders. Next, consideration of the lead author’s primary affiliation status may have also underestimated the results; it is not uncommon for many authors to have multiregional affiliations. Last, estimates of regional disease burden and population prevalence ratios used multiple data sources, and these should be interpreted with caution and considering local evidence.

Despite modest improvements and favorable trends over the last decade, APAC participants and authors remain significantly under-represented in seminal cardiometabolic trials. Barriers to design, conduct, funding, and leadership in trials in the APAC region call for a multipronged approach: 1) development and integration of research participant’s diversity and inclusivity action plan into the good clinical practice guidelines for sponsors and investigators undertaking phase 3 clinical trials; 2) systemic and concerted efforts by regulatory agencies, funders, and investigators to address several upstream (limited capacity of sites to conduct trials per U.S. Food and Drug Administration standards, political economy of participation in foreign funded trials) and downstream factors (limited awareness of benefits in trial participation, fear of side effects with new treatment) negatively affecting Asian participation in trials; 3) greater investment in training and capacity-building programs for early- to mid-career clinicians focused not only on research methodology but also scientific leadership skills (eg, the COALESCE program in India^9^); 4) increased global solidarity and funding support from the local government to promote APAC researcher-led clinical trials; and 5) pledge by researchers and other stakeholders to improve reporting of race/ethnicity and region-specific recruitment goals to enhance the generalizability of trial results.

In conclusion, the enrollment of Asian/APAC participants in global CMD trials is poor, but improving. In an era of data-driven medicine, improving what we do not measure is difficult. Clear and consistent reporting and diverse participation of race/ethnicity represent an achievable goal that enables downstream innovation in research practice and accountability. Information tools embedded within clinical trials registry to analyze race/ethnicity data could allow researchers to examine the trend (cross-tabulation of race/ethnicity by registered trials) and learn from similar trials while anticipating likely challenges to diverse recruitment. Compulsory reporting of race/ethnicity for funding or journal publication could also be effective in boosting Asian/APAC representation. Additional incentives and enforced regulations may be needed to ensure trial sponsors are engaged and accountable for recruiting diverse participants in clinical trials. All stakeholders (researchers, academia, industry, government funders, patient advocacy groups, and regulatory authorities) must commit to recruiting diverse, representative populations and consistent and transparent reporting of race/ethnicity to enable innovative solutions to global health problems.

## Funding Support and Author Disclosures

The authors have reported that they have no relationships relevant to the contents of this paper to disclose.

## References

[1] Miranda J.J., Barrientos-Gutierrez T., Corvalan C. (2019). Understanding the rise of cardiometabolic diseases in low- and middle-income countries. Nat Med.

[2] Flood D., Seiglie J.A., Dunn M. (2021). The state of diabetes treatment coverage in 55 low-income and middle-income countries: a cross-sectional study of nationally representative, individual-level data in 680 102 adults. Lancet Healthy Longev.

[3] Qureshi N.Q., Mufarrih S.H., Bloomfield G.S. (2021). Disparities in cardiovascular research output and disease outcomes among high-, middle- and low-income countries - an analysis of global cardiovascular publications over the last decade (2008-2017). Glob Heart.

[4] Williams R., Karuranga S., Malanda B. (2020). Global and regional estimates and projections of diabetes-related health expenditure: results from the International Diabetes Federation Diabetes Atlas, 9th edition. Diabetes Res Clin Pract.

[5] Khunti K., Bellary S., Karamat M.A. (2017). Representation of people of South Asian origin in cardiovascular outcome trials of glucose-lowering therapies in Type 2 diabetes. Diabet Med.

[6] Sawant S., Wang N. (2023). Underrepresentation of ethnic and regional minorities in lipid-lowering randomized clinical trials: a systematic review and meta-analysis. Eur J Prev Cardiol.

[7] Vilcant V., Ceron C., Verma G., Zeltser R., Makaryus A.N. (2022). Inclusion of under-represented racial and ethnic groups in cardiovascular clinical Trials. Heart Lung Circ.

[8] Azzopardi R., Nicholls S.J., Nerlekar N. (2023). Asia-Pacific investigators and Asian enrollment in cardiometabolic trials: insights from publications between 2011 and 2020. JACC: Asia.

[9] COllaborative Research Implementation, And LEadership Training to AddresS Chronic Conditions across the Life CoursE (COALESCE) program. https://scholarblogs.emory.edu/coalesce/program-overview/.

